# The Effect of Supplementation Using a Mixture of Fish Oil and Linseed on the Level of Immunomodulatory Components in Bovine Colostrum

**DOI:** 10.3390/molecules28052154

**Published:** 2023-02-24

**Authors:** Kinga Grodkowska, Marcin Gołębiewski, Jan Slósarz, Tomasz Sakowski, Kamila Puppel

**Affiliations:** 1 Institute of Animal Science, Warsaw University of Life Sciences, Ciszewskiego 8, 02-786 Warsaw, Poland; 2 Institute of Genetics and Animal Biotechnology, Polish Academy of Science, Jastrzębiec, Postępu 36A, 05-552 Magdalenka, Poland

**Keywords:** colostrum, immunoglobulins, fish oil and linseed supplementation, calves, immunity

## Abstract

The aim of this study was to determine the effect of supplementing rations, with a mixture of fish oil and linseed, on the level of immunomodulatory components in colostrum. Twenty multiparous cows, that were three weeks before scheduled calving, had a body condition of 3–3.5, and had not been diagnosed with multiple pregnancies, were qualified for the experiment. The cows were divided into two groups: experimental (FOL) (n = 10) and control (CTL) (n = 10). The CTL group were individually given the standard food ration for dry cows for about 21 days before calving, while the FOL group received food rations that were enriched with 150 g of fish oil and 250 g of linseed (golden variety). Colostrum samples for testing were taken twice a day on the first and second days of lactation, and then once a day from the third to the fifth day of lactation. The experiment showed that the applied supplementation had an impact, in the form of increasing the fat, protein, IgG, IgA, IgM, vitamin A, C22:6 n-3 (DHA), and C18:2 cis9 trans11 (CLA) contents in colostrum; however, the C18: 2 n-6 (LA) and C20:4 n-6 (AA) contents decreased. Due to the lower quality of colostrum found in high-yield cows, and therefore in the Holstein–Friesian breed, it is possible to improve the quality by, among other things, introducing nutritional modifications during the second stage of the dry period.

## 1. Introduction

With the current intensive use of dairy cattle, it is extremely important to ensure the calves’ highest possible survival rate, as well as their rapid and healthy rearing. Calves come into the world having only developed non-specific immunity, which is associated with the activity of phagocytes, granulocytes, and the complement system’s activity. The development of a specific, active immunity occurs after contact with pathogens and the development of appropriate defense mechanisms. This is due to the peculiar structure of the syndesmochorial placenta, which inhibits completely the transfer of immune proteins between the cow’s organism and the calf’s. Therefore, humoral passive immunity can only be established through the delivery of good-quality colostrum, which is a source of antibodies [1,2].

The process of colostrogenesis begins about 21 days before calving. Due to an increase in the concentration of lactogenic hormones (estrogen, progesterone, prolactin), the active transfer of immunoglobulins (Ig) from the blood to the mammary gland occurs. The main immunoglobulin transported is IgG1, the concentration of which is up to ten times higher than IgG2, in colostrum. Besides immunoglobulins, colostrum also contains other components necessary for the newborn calf, such as growth factors, leukocytes, cytokines, hormones, and nutrients. Compared to milk, colostrum has a significantly higher density and concentration of bioactive components, in both the protein fraction (including immunoglobulins), and the fat fraction (polyunsaturated fatty acids, fat-soluble vitamins) [3,4,5]. After labor, the process of colostrogenesis ceases, which leads to a gradual change in the concentration of the various components, and results in the secretion becoming milk [6,7,8].

Colostrum should be given to the calf as soon as possible (0.5–2 h) after birth, so that all the components of colostrum can be properly absorbed. During the first hours of a calf’s life, proteolytic enzymes in its digestive system are inactive. This allows for the multistage absorption of intact proteins from the gastrointestinal tract, which, based on the pinocytosis principle, then pass into the mucosa of the small intestine and then into the lymph [2,9]. The most important components in colostrum are immunomodulators. These have the greatest effect on altering the body’s immune response. Their effect can either be one of enhancement or inhibition in relation to the components of the immune system [10]. Immunomodulatory components in the protein fraction include immunoglobulins, lactoferrin (LF), lysozyme (Lz), and the albumins β-lactoglobulin (β-LG) and α-lactalbumin (α-LG). Igs are bioactive immune proteins that determine the body’s humoral response. They act through phagocytosis, can bind antigens, and have the ability to activate the complement system. Immunoglobulins of different classes can be distinguished in colostrum: IgG, IgA, IgM, and IgE [11]. The transport of Ig from the maternal bloodstream to the mammary gland is carried out by specific receptors that capture IgG from the intercellular fluid, transport it across the barrier, and then release it into the lumen of the gland. This transfer can be inhibited by an increase in concentration of prolactin in the cow’s blood during the first days of lactation [12]. Both α-LG and β-LG belong to the whey protein group, and are found in colostrum and milk. In colostrum, the concentration of β-LG is about 14 mg/mL, which is almost three times higher than in milk [13,14]. β-LG is resistant to the acids and proteolytic enzymes present in the stomach. As a result, it can be absorbed through the intestinal wall during the first days of a calf’s life [15]. The tertiary structure shows strong homology with the retinol-binding plasma protein, and other proteins involved in the transport of small, hydrophobic molecules. This translates into it taking part in the transport of retinol and fatty acids, and the compounds dissolved in them. In addition, β-LG binds cholesterol and vitamin D, and stimulates lipase activity [15]. The immunomodulatory activity of β-LG has also been demonstrated. Another important bioactive constituent of colostrum is lactoferrin (LF). It is a glycoprotein of the transferrin family, which has the ability to actively uptake iron ions in the intestine. Receptors for lactoferrin are found in intestinal mucosa, monocytes, macrophages, neutrophils, lymphocytes, and platelets [16,17]. In addition to contributing to maintaining homeostasis in iron metabolism, LF protects the organism from the growth of pathogenic microorganisms: bacteria, fungi, and viruses. It also has immunomodulatory and anti-inflammatory effects, and participates in the process of cell differentiation [18]. LF restricts the growth of both Gram-positive and Gram-negative bacteria (e.g., *Escherichia coli*, *Salmonella typhimurium*, *Shigella dysenteriae*, *Listeria monocytogenes*, *Streptococcus mutans*, *Bacillus stearothermophilus*, and *Bacillus subtilis*). Fungicidal activity has also been observed, especially against *Candida* spp. [19]. LF’s modulation of the immune response occurs by activating the complement system, influencing phagocyte activity, accelerating the maturation of T-lymphocyte precursors, and differentiating immature B-lymphocytes. In addition, LF catalyzes the conversion of the inactive form of lysozyme into its active form, and exhibits a synergistic effect with respect to this form [10,16,19]. Lysozyme (LZ) is an enzymatic protein of the hydrolase group that exhibits lytic activity. Among other things, it is present in body fluids (including colostrum) and phagocytes—neutrophils and macrophages—making it a component of the non-specific immunity mechanisms [16]. Like lactoferrin, it has antibacterial, antifungal, and antiviral properties. LZ is active mainly against Gram-positive bacteria (e.g., *Bacillus cereus*, *Clostridium butyricum*, *Clostridium tyrobutyricum*, *Streptococcus faecalis*, *Micrococcus*, and *Pseudomonas fluorescens*) [16,20]. LZ shows little hydrolytic activity against Gram-negative bacteria, due to the presence of additional lipoproteins, liposaccharides, and polypeptides on the bacteria’s cell wall surface, limiting the enzyme’s ability to penetrate it [21].

Another important group of colostrum bioactive compounds are fatty acids. In addition to providing energy, they have an immunomodulatory function. Lipids build the cell’s membrane and ensure its integrity; moreover, they are the precursors of biologically active compounds, for example, hormones, which provide a condensed source of energy and are carriers of the fat-soluble vitamins A, D, E, and K [22]. Fatty acids obtained from food can promote or inhibit the body’s immune response, and enhance or attenuate the development of inflammation. This effect depends on the profile of the fatty acids obtained, their amount, and their ratios to each other [23]. A distinction is made between saturated (SFA) and unsaturated fatty acids (i.e., monounsaturated (MUFA) and polyunsaturated (PUFA)) [22]. PUFA n-3 is a group of polyunsaturated fatty acids whose first double bond is located at the third carbon atom, counting from the methyl group. This group of fatty acids includes α-linolenic acid (ALA) (C_18_H_30_O_2_), eicosapentaenoic acid (EPA) (C_20_H_30_O_2_), and docosahexaenoic acid (DHA) (C_22_H_32_O_2_). The metabolic transformation of ALA leads to the formation of EPA and DHA [24]. Products that have high levels of omega-3 fatty acids are fish, oils extracted from fish, and some plant seeds, such as chia and sesame seeds. Among fish oils, the highest levels of n-3 fatty acids are found in oils from herring, salmon, sardines, and menhaden, as well as cod liver oils [25]. PUFA n-6 is a group of long-chain polyunsaturated fatty acids whose first double bond is located at the sixth carbon atom, counting from the methyl group. This family of fatty acids includes, among others, linoleic acid (LA) (C_18_H_32_O_2_) and arachidonic acid (AA) (C_20_H_32_O_2_). Products that have high levels of omega-6 fatty acids are oilseeds and the oils obtained from them. The highest levels are found in soybean, sunflower, sesame, rapeseed, and corn oils [25,26]. Conjugated linoleic acid (CLA) is a group of linoleic acid isomers. They occur naturally in beef and milk, but can also be produced by hydrogenation. Twenty-eight CLA isomers have been identified in milk. The isomer found in the greatest quantity is cis9, trans11-CLA, which accounts for up to 90% of total CLA. It is to this isomer that the most beneficial properties are attributed [27]. The immunomodulatory effect of CLA involves inhibiting cell proliferation in T cells, and increasing the expression of interleukin-2 (IL-2) and interferon-γ (IFN-γ), which play important roles in the innate and adaptive immune responses; for example, IL-2 is a potent T-cell growth factor [28,29]. CLA has the ability to reduce the production of pro-inflammatory prostaglandins such as TNF-α, PGE2, and IL-1β, for example. In addition, CLA can inhibit inflammatory responses of macrophages [27]. However, the effect of CLA on Ig concentrations is unclear. A study by Ramirez-Santana et al. [30] showed that CLA supplementation increased serum IgG, IgA, and IgM concentrations in young rats, while a study by Hussen et al. [31] showed a reduction in serum and milk IgG concentrations in CLA-supplemented cows. After absorption, fatty acids undergo a series of enzymatic transformations that take place in the cell’s endoplasmic reticulum. Eicosanoids, which are a group of chemical messengers that act within the immune system, are synthesized from PUFAs. Eicosanoids include prostaglandins, thromboxanes, leukotrienes, and lipoxins, among others [32,33]. Eicosanoids regulate the immune system by affecting cytokine production, cell differentiation and proliferation, and antibody formation. They have local effects, interacting with membrane receptors. Depending on the physiological state of the organism, the immune cell population, and other acting factors, eicosanoids can elicit different responses from the body—both immunomodulatory and immuno-inhibitory; however, a pro-inflammatory effect is more often observed. PGs and LTs have the strongest pro-inflammatory effects. PGs increase the sensation of pain and inflammation. They can affect intestinal peristalsis, causing diarrhea. Depending on the type, they also affect vasodilation or vasoconstriction, and counteract platelet aggregation [32,33].

Colostrum and cow’s milk are rich sources of fat-soluble vitamins. Vitamins belong to a group of organic chemical compounds, and are essential for maintaining the body’s homeostasis. Vitamins have antioxidant properties, are involved in biochemical transformations as coenzymes, and can be the precursors of hormones. The precursor of vitamin A is β-carotene. It is not degraded in the rumen; but in the intestinal mucosa, it is converted to retinol, which is absorbed and transported to the liver [34]. Vitamin A exists in the form of several different compounds: retinol, retinal, retinoic acid, 3,4-didehydroretinol, and 3,4-didehydroretinoic acid [35]. Vitamin A influences the proper differentiation of cells, and the healthy course of pregnancy and development of the body; and is involved in vision and immune processes (mainly in the course of infectious diseases). In addition, vitamin A, like vitamin E, has antioxidant properties—it has the ability to react with superoxide free radicals, thus protecting lipids from peroxidation. The α-tocopherol form of vitamin E has the ability to increase the efficiency of neutrophils, and protects them from oxidative damage after phagocytosis and bacterial lysis [34,35,36].

The quality of colostrum is influenced not only by high levels of immunoglobulins, but also by the concentrations of other bioactive biochemical compounds that stimulate the maturation and function of the newborn’s digestive tract, and perform bactericidal and virucidal functions. Puppel et al. [37] showed a significant influence of colostrum quality class on the formation of the intestinal microflora and the daily weight gains of calves. The higher the concentration of bioactive components, the more probiotic bacterial strains can develop. The content level of all components in colostrum is variable, and depends on many factors. There are both genetic factors such as breed, milk yield, and parity; and environmental factors such as housing systems and nutrition. Additionally, more than 60% of colostrum samples have reduced immunological quality. Therefore, new solutions are increasingly being sought to increase the level of bioactive components in colostrum. The aim of this study was to determine the effect of supplementing rations, with a mixture of fish oil and linseed, on the level of immunomodulatory components in colostrum.

## 2. Results

The experiment showed that supplementation with a mixture of fish oil and linseed had a significant effect on the formation of the colostrum’s basic chemical composition. When comparing CTL and FOL, it was found that the concentrations of protein, fat, and casein were higher in the FOL group for all draws analyzed, except for intake 1a, where casein content was higher in the control group. Casein content was highest for the first two intakes for both the experimental group and the control group.

**Figure 1:**
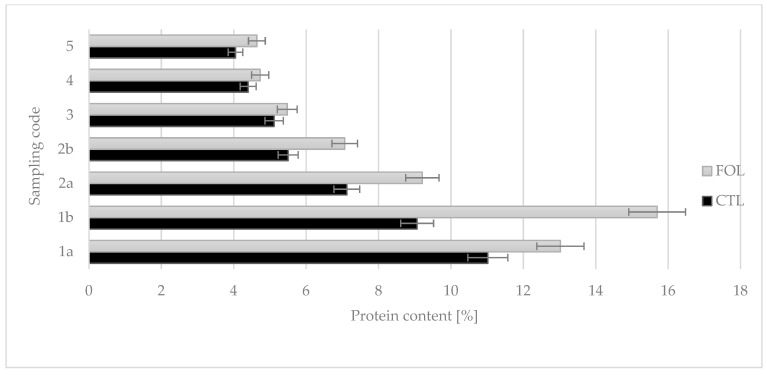
Comparison of the effect of supplementation on colostrum protein content. CTL received a standard ration for dry cows, FOL received a ration enriched with 150 g of fish oil and 250 g of linseed. Data are presented as least squares means with a standard error of the mean. Statistical differences between groups (CTL vs. FOL) at *p* ≤ 0.05, and collections at *p* ≤ 0.01.

The colostrum from supplemented cows had a significantly higher protein concentration (*p* ≤ 0.01). The average protein content for the FOL group was 8.55%, while for the CTL group it was 6.61%. The differences in protein concentration were particularly noticeable in the first and second intakes: respectively, 13.023% and 15.698% for the FOL group, and 11.019% and 9.066% for the CTL group (Figure 1).

The applied supplementation also had a beneficial effect on the fat content of colostrum. The colostrum from the supplemented cows contained 32.73% more fat than that of the control group, while the average fat content in colostrum was 5.67% in the FOL group and 4.27% in the CTL group.

**Figure 2:**
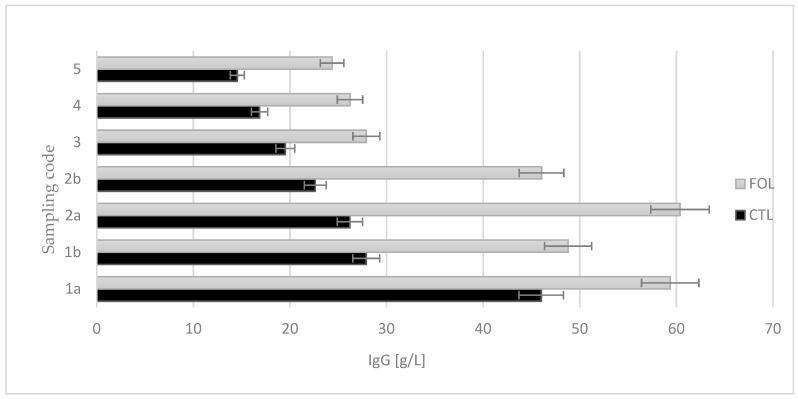
Comparison of the effect of supplementation on the immunoglobulin class G content in colostrum [g/L]. CTL received a standard ration for dry cows, FOL received a ration enriched with 150 g of fish oil and 250 g of linseed. Data are presented as least squares means with a standard error of the mean. Statistical differences between groups (CTL vs. FOL) at *p* ≤ 0.01, and collections at *p* ≤ 0.01.

The colostrum collected from the cows assigned to the experimental group, during the first and second intakes, had a statistically significant (*p* ≤ 0.01) higher concentration of IgG (59.358 g/L and 48.78 g/L, respectively) compared to the control group (46.011 g/L and 27.889 g/L, respectively) (Figure 2). The average concentration of IgG was 41.86 g/L for the study group, and 24.80 g/L for the control group.

**Figure 3:**
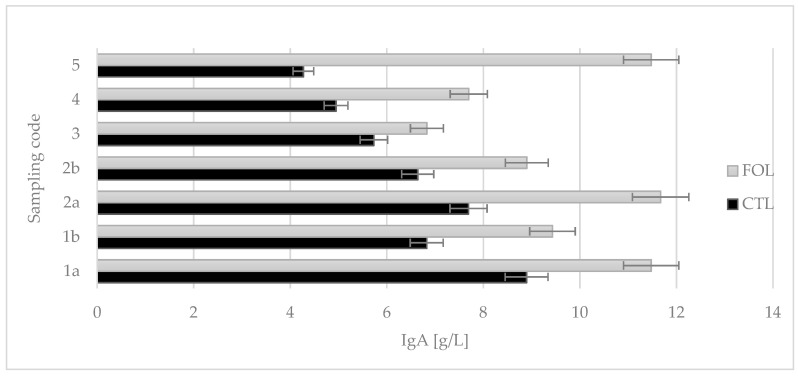
Comparison of the effect of supplementation on the immunoglobulin class A content in colostrum. CTL received a standard ration for dry cows, FOL received a ration enriched with 150 g of fish oil and 250 g of linseed. Data are presented as least squares means with a standard error of the mean. Statistical differences between groups (CTL vs. FOL) at *p* ≤ 0.01, and collections at *p* ≤ 0.01.

Colostrum assigned to the FOL group from the first intake had a significantly higher concentration of IgA (11.476 g/L) compared to the CTL group’s first intake (8.895 g/L). The study showed that the supplementation used had a statistically significant (*p* ≤ 0.01) effect on the Ig A levels (Figure 3).

**Figure 4:**
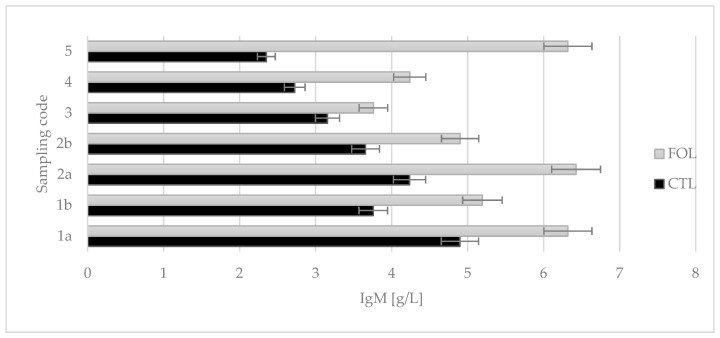
Comparison of the effect of supplementation on the class M immunoglobulin content in colostrum. CTL received a standard ration for dry cows, FOL received a ration enriched with 150 g of fish oil and 250 g of linseed. Data are presented as least squares means with a standard error of the mean. Statistical differences between groups (CTL vs. FOL) at *p* ≤ 0.01, and collections at *p* ≤ 0.01.

Colostrum assigned to the FOL group from the first intake had a statistically significant higher concentration of IgM (6.319 g/L) compared to the CTL group’s first intake (4.898 g/L). The study showed that the supplementation used had a statistically significant (*p* ≤ 0.01) effect on the Ig M levels (Figure 4).

**Figure 5:**
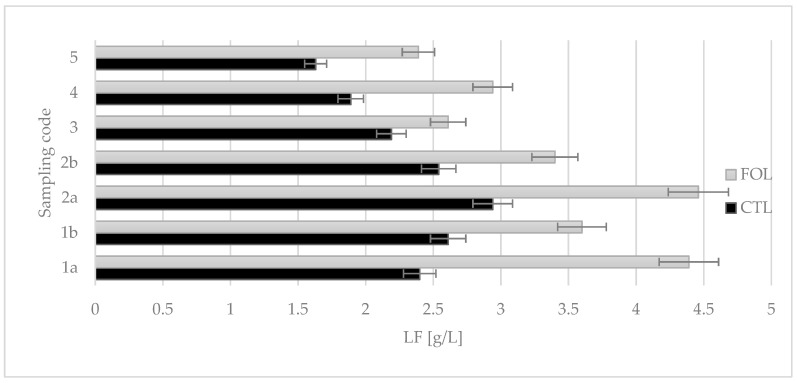
Comparison of the effect of supplementation on LF content in colostrum. CTL standard received a ration for dry cows, FOL received a ration enriched with 150 g of fish oil and 250 g of linseed. Data are presented as least squares means with a standard error of the mean. Statistical differences between groups (CTL vs. FOL) at *p* ≤ 0.01, and collections at *p* ≤ 0.01.

Lactoferrin content in the colostrum assigned to the FOL group was statistically significantly higher (*p* ≤ 0.01) compared to the control group. The highest value was observed for intakes 1a and 2a (Figure 5): 4.46 g/L and 4.39 g/L, respectively. The concentration of LF in the control group did not exceed 3.0 g/L, while the average content was 2.31 g/L. In the experimental group, the average LF content was 3.39 g/L. The lowest LF concentration for the FOL group was not lower than 2.39 g/L.

In the performed experiment, the supplementation reduced the content of lysozyme in colostrum. The highest LZ content in colostrum, for both the FOL and CTL groups, was found for intake 1b: 834.06 mg/L and 945.26 mg/L, respectively. The average LZ content in colostrum assigned to the FOL group was 643.86 mg/L.

**Figure 6:**
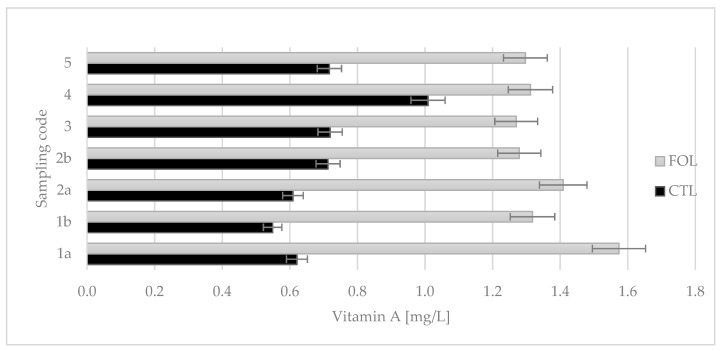
Comparison of the effect of supplementation on vitamin A content in colostrum. CTL received a standard ration for dry cows, FOL received a ration enriched with 150 g of fish oil and 250 g of linseed. Data are presented as least squares means with a standard error of the mean. Statistical differences between groups (CTL vs. FOL) at *p* ≤ 0.01, and collections at *p* ≤ 0.01.

The experiment showed that supplementation with flaxseed and flaxseed oil significantly increased the vitamin A content of colostrum (Figure 6). The average content of vitamin A in colostrum from the FOL group (1.35 mg/L) was almost double that of the control group (0.70 mg/L).

The vitamin E content of the colostrum from the FOL group was higher compared to the CTL group. However, no statistically significant differences were shown. In the FOL group, the vitamin E content did not exceed 1.53 mg/L. In the conducted experiment, colostrum assigned to the FOL group had a higher average vitamin D content during the first and second days of lactation (8.98 μg/L and 10.85 μg/L, respectively) compared to the CTL group (7.19 μg/L and 5.96 μg/L, respectively). In addition, the total average vitamin D concentration from all intakes was 23.63% higher for the FOL group.

**Figure 7:**
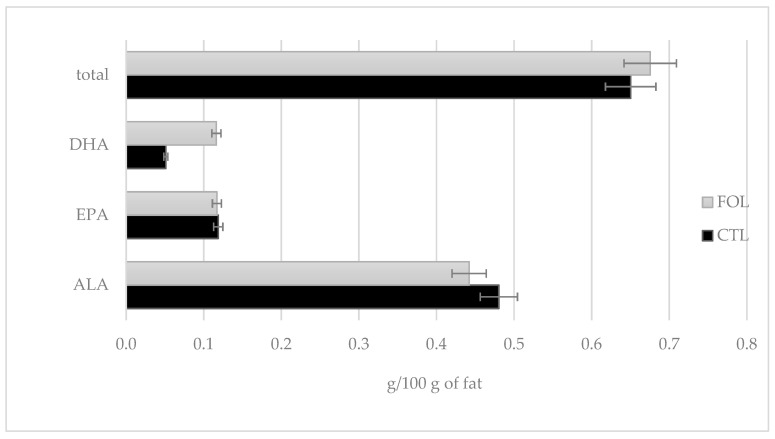
Comparison of the effect of supplementation on PUFA n-3 content in a pool of seven colostrum samples, taken between day 1 and day 5. CTL received a standard ration for dry cows, FOL received a ration enriched with 150 g of fish oil and 250 g of linseed. Data are presented as least squares means with a standard error of the mean. Statistical differences between groups (CTL vs. FOL) at *p* ≤ 0.05.

In the performed experiment, the supplementation significantly affected the DHA content in colostrum (*p* ≤ 0.01). At its peak, the concentration of DHA in colostrum from the FOL group was 0.207 g/100 g fat. Colostrum assigned to the FOL group had more than twice the average DHA content relative to the CTL group. The ALA content for the FOL group was 8% lower than for the CTL group. No effect of the supplementation was observed on EPA content (Figure 7).

**Figure 8:**
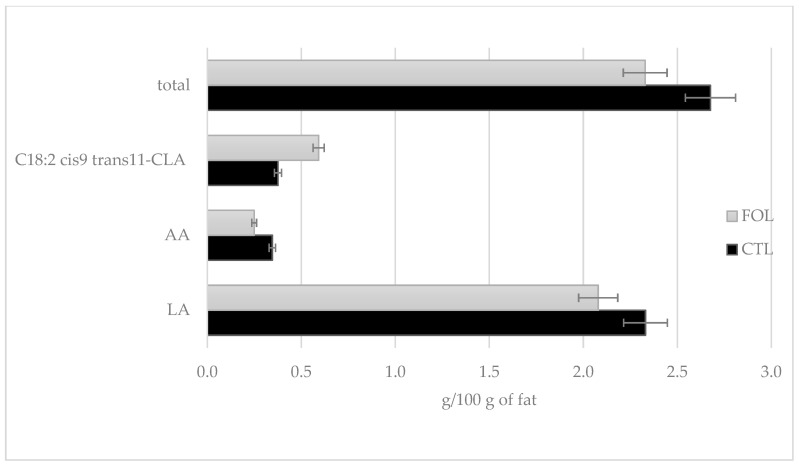
Comparison of the effect of supplementation on PUFA n-6 in a pool of seven colostrum samples, taken between day 1 and day 5. CTL received a standard ration for dry cows, FOL received a ration enriched with 150 g of fish oil and 250 g of linseed. Data are presented as least squares means with a standard error of the mean. Statistical differences between groups (CTL vs. FOL) at *p* ≤ 0.05.

In the performed experiment, the applied supplementation had a significant (*p* ≤ 0.01) effect on increasing the content of C18:2 cis9 trans11-CLA. The maximum CLA concentration in the FOL group was 0.777 g/100 g fat. The average CLA content in the FOL group was 57.42% higher compared to the CTL group. The supplementation used reduced the average AA and LA contents by 38.68% and 12.06%, respectively (Figure 8). Compared to the CTL group, the PUFA content in the FOL group decreased, but the colostrum from the FOL group had a better n-3:n-6 acid ratio: 1:4 in the FOL group, and 1:5 in the CTL group. The saturated fatty acid (SFC) content of the FOL group’s colostrum was 2.02% lower than that of the CTL group. The difference was not statistically significant.

## 3. Discussion

Colostrum samples for testing were taken twice a day on the first and second days of lactation (1a, 1b and 2a, 2b), and then once a day from the third to fifth (3, 4, and 5) days of lactation. Sampling colostrum in this way made it possible to precisely track changes in the levels of the colostrum’s bioactive components over time. The composition of colostrum changes from hour to hour, and the biological and nutritional value also decreases (Figure 1, Figure 2, Figure 3, Figure 4, Figure 5 and Figure 6). Thus, delaying the calf’s first and subsequent feedings of colostrum causes it to benefit less from its nutritional and health benefits. Absorption of intact proteins from the gastrointestinal tract takes place in stages. Initially, by pinocytosis, proteins pass into the mucosa of the small intestine and then into the lymph. Therefore, it is important to give colostrum to the calf as soon as possible after birth (0.5–2 h). As early as the 6th hour after calving, the possibility of absorption of antibodies from colostrum is reduced by a third, after 12 h it is already reduced by two-thirds, and after 24 h, the intestinal barrier appears [9]. The study showed that the experimental group receiving LC showed a significantly lower reduction in immunoglobulin concentration over the course of the experiment (Figure 2, Figure 3 and Figure 4). A similar relationship was also shown for the formation of fatty acids and fat-soluble vitamins in the LC group, and collection 1a, 1b, 2a, 2b, 3, 4, and 5 (Figure 6, Figure 7 and Figure 8).

The immunomodulatory content of colostrum is influenced by many factors. Literature data show significant differences in IgG concentrations depending on the breed: Simmental—5799 mg/dL; Jersey—5232 mg/dL; Holstein–Friesian—4699 mg/dL [8,11,38]. In addition, in an experiment [39] conducted on eight Jersey and eight PHF cows, it was shown that colostrum from the Jersey cows contained higher levels of α-LA and LF, especially during the first two days of lactation. The concentration of β-LG was comparable for both analyzed breeds.

There have also been differences observed in the levels of immunomodulatory components in colostrum depending on lactation number. In one experiment [8], it was observed that colostrum from primiparous cows had about 5% lower IgG content compared to colostrum from multiparous cows. This experiment confirmed results obtained by Bar et al. [40]. The effect of lactation number on the level of protein-fraction immunomodulatory components has also been observed in milk. Król et al. [41] showed an increase in IgG, LZ, and LF contents for successive lactation numbers. In addition, calving season may also affect the levels of immunomodulatory components in colostrum. However, the data available in the literature are inconclusive. Experiments conducted by Gulliksen et al. [42] and Abdullahoğlu et al. [43], showed a higher immunoglobulin content in the colostrum of cows who calved during spring and summer, while experiments conducted by Dunn et al. [44] and Genc and Coban [45], showed the opposite relationship.

A correlation has also been observed between the occurrence of inflammation of the mammary gland (mastitis) and a decrease in the quality of the colostrum secreted. As reported by Puppel et al. [9], this influence is not yet clearly defined, due to diverging results, but the available literature suggests that mastitis contributes to a decrease in colostrum yield, density, and protein content [46,47]. In order to avoid the above-mentioned factors influencing the experiment, healthy animals (without known metabolic diseases), of one breed (HF), and of similar ages (2nd or 3rd lactation) were selected. This selection for the study group allowed a more accurate assessment of the effect of supplementation on colostrum quality, as the other factors were eliminated.

An important aspect affecting the quality of produced colostrum is the nutrition during the dry period. The optimal drying-out period for cows lasts from 40 to 60 days. This is the time when the cow’s body prepares to enter the next lactation. There are two stages in the dry period: the proper dry period (from 7–8 weeks before calving to 3 weeks before calving) and the transition period (lasting 21 days before calving) [48]. Feeding cows during the transition period may affect the immunoactive content of colostrum, because it is during this time that colostrogenesis takes place. This assumption is supported by studies. An experiment conducted by Nowak et al. [49] showed that lower levels of nutrition during the pre-calving period had a significant effect in increasing IgA content in colostrum. Also, an experiment conducted by Mann et al. [50] showed the beneficial effect of lower feeding levels on the immunoglobulin content of colostrum. A study performed by Jolazadeh et al. [51] showed that supplementation using Ca^2+^ salt from soybean oil, and Ca^2+^ salt from fish oil, three weeks before scheduled calving, had a positive effect on the n-3 and n-6 fatty acid contents in colostrum. The addition of pumpkin and synthetic β-carotene significantly increased the level of immunoglobulins in colostrum. Lysozyme content was also significantly higher in cows fed fodder supplemented with pumpkin or synthetic β-carotene [52].

The most prominent among all immunoglobulins are class G (about 85–90%), of which almost 90% are IgG1. The average IgG, IgA, and IgM concentrations are 47.6, 4.2, and 3.9 mg/mL, respectively [11,53]. The concentration of Ig type G should be higher than 50 g/L [54]. 

Immune proteins are absorbed during the first 72 h after birth. The most effective process occurs in the first two to four hours of life. Even as little as six hours after birth, the calf’s capacity for absorbing immunoglobulins is only 50%, and after 12 h it begins to decrease more and more rapidly. Stimulation of epithelial cells, using other types of food, further reduces the calf’s capacity for absorbing immunoglobulins, which is why it is so important to give colostrum to calves as soon as possible. Properly developed calves, that had an easy parturition and that have high vitality, are able to drink colostrum as early as the first hour of life [2,55,56]. Confronting the results obtained in the literature data, colostrum from intakes 1a and 2a from cows in the FOL group was characterized by a concentration of IgG ≥ 50 g/L, and thus met the requirements for high immunological quality, which guarantees adequate passive transfer. Colostrum from cows in the control group contained < 50 g/L IgG, and thus could be considered poor quality colostrum.

Lactoferrin has the ability to modulate the immune response. The average LF content in the cows’ colostrum depends on the breed, duration of lactation, and lactation number, but can be 1.5–5 g/L, while in mature milk it is about 0.1 mg/mL [16]. The proposed supplementation favorably increased the LF content in colostrum. The LF content in the samples of the research group reached the upper values in relation to the literature data. In an experiment conducted by Puppel et al. [57] on the effect of supplementation, with a mixture of flaxseed and fish oil, on the protein composition of cows’ milk, an increase in LF content after 21 days of supplementation was also demonstrated.

The LZ content of the tested samples was lower compared to the control group. This could have been related to the improvement of the milk’s somatic parameters. The average level of lysozyme in the samples of the FOL group was comparable with the literature data, according to which the content of LZ in colostrum is 140–700 mg/L [16].

The placenta of ruminants limits the transfer of vitamin A and vitamin E. Colostrum that has high concentrations of these vitamins can prevent the occurrence of deficiencies in these vitamins and associated diseases. If a cow’s feed ration during pregnancy, especially during colostrogenesis, is deficient in feed containing high amounts of vitamins A and E, their supply to the calf (transfer from cow’s to calf’s system) may be insufficient [36].

Despite almost double the average vitamin A content in the FOL group (1.35 mg/L) compared to the CTL group (0.71 mg/L), when compared to the literature data, the results are not high concentrations. According to the literature data, the average concentration of vitamin A in colostrum is 2.33–3.69 mg/L [4,5,36]. This indicates that it is reasonable to use supplementation to increase the vitamin A content in the colostrum of cows on the farm where the experiment was conducted. Omega-3 and omega-6 fatty acids share common metabolic steps, making them compete for the same substrates. Excess LA results in impaired ALA metabolism and reduced incorporation of ALA into cellular phospholipids. The results of LA metabolism are arachidonic acid and OXLAMSs (oxidized linoleic acid metabolites), which have been linked to pathological conditions in the body [58].

Fatty acids in colostrum and milk play an important nutritional and immunomodulatory role. In particular, PUFAs have antioxidant abilities, and reduce the activity of pro-inflammatory mediators [59]. As reported by Opgenorth et al. [60], administering colostrum enriched with flaxseed and fish oil, reduced oxidative stress in calves. This suggests a potential benefit in increasing the anti-inflammatory status of calves. While, a study by Śpitalniak-Bajerska et al. [61] showed that the addition of flaxseed oil to milk replacer had a positive effect on calf health, weight gain, growth rate, and feeding efficiency. In the context of this information, it seems important to increase the unsaturated fatty acid content of colostrum. The results obtained partially correspond with those obtained by Mašek et al. [62]. Their study consisted of administering a supplement with high EPA and DHA contents to Simmental cows for a period of 14 days before scheduled calving: a statistically significant increase in ALA (*p* ≤ 0.01) and EPA (*p* ≤ 0.05) content was found. No significant differences were shown for DHA content. The total n-3 acid content was significantly higher (*p* ≤ 0.001) for the control group. Also, a study by Leiber et al. [63] showed that a concentrate with high flaxseed content had a significant effect in increasing ALA in the first days of lactation after supplementation. A positive effect on increasing EPA and DHA in the colostrum of cows was shown by Jolazadeh et al. [50], after supplementation with Ca salts from fish oil.

The effect of fish oil supplementation on the fatty acid profile of colostrum has also been studied in other ruminant species. An experiment by Cattaneo et al. [64], conducted on goats, showed significant increases in EPA and DHA after supplementation, and a decrease in ALA. Also, in a study conducted by Annett et al. [65], on sheep, there were significant (*p* ≤ 0.001) increases in the EPA and DHA contents in colostrum after fish oil supplementation. The results obtained in the current study are in opposition to those obtained in the previously cited study by Mašek et al. [62]. The levels of AA and LA were significantly (*p* ≤ 0.001) higher in the experimental group relative to the control group. In a study by Santschi et al. [66], one of the experimental groups received a feed supplement whose content was made up of 50% extruded flaxseed, for four weeks before scheduled calving. Colostrum from these cows had significantly higher (*p* ≤ 0.05) ALA and CLA contents. No significant differences were found for EPA and LA contents. Jolazadeh et al. [51] showed that after supplementation with Ca salt from fish oil, there was no significant effect on LA or CLA content. The previously cited study by Cattaneo et al. [64] showed that applied supplementation had the effect of reducing LA content in goat colostrum. A similar result was obtained by Annett et al. [65].

## 4. Materials and Methods

### 4.1. Study Site and Study Animals

The experiment was conducted on a high-input farm, using 350 Polish Holstein–Friesian cows, of the black and white type, which were fed year-round with total mixed ration (TMR). The animal study protocol was approved by the Second *Ethics Committee for Animal Experimentation in Warsaw* (protocol number WAWA2/086/2018). The conditions for including animals in the experiment were that they had not been recorded as having multiple pregnancies, had a body condition score (BSC) of 3–3.5 points, and were multiparous in second or third lactation three weeks before scheduled calving. Two groups were created for the experiment: control (n = 10) and experimental (n = 10). The control group (CTL) received the standard ration for dry cows, while the experimental group (FOL) received a ration enriched with 150 g of fish oil and 250 g of golden linseed (Table 1). The supplementation was administered for a period of about 21 days, individually, to each cow in the feedlot (until calving). The first sample of colostrum was collected up to two hours after calving. Colostrum samples for testing were taken twice a day on the first and second days of lactation (1a, 1b and 2a, 2b), and then once a day from the third to the fifth (3, 4 and 5) day of lactation. The cows were fully milked at each milking, the colostrum yield was medium (4–7 kg) for all cows, and foremilk was stripped out of the gland prior to sample collection.

Dry cows were fed according to the guidelines of the Nutrient Requirements Committee (Table 2).

### 4.2. Chemical Analyses of Colostrum

Basic chemical composition: fat, total protein, lactose, dry matter, non-fat dry matter, casein, freezing point, density, citric acid, urea, free fatty acids, and acidity, were determined using a MilkoScan FT 120, from Foos Electric.

The determination of whey proteins was by RP-HPLC chromatography (Series 1100; Agilent Technologies Waldbronn, Germany), according to the methodology described by Puppel et al. [67]. Separations were performed at ambient temperature, using solvent gradient with a C18 300A Jupiter column (Phenomenex, Torrance, CA, USA). The identification of peaks was confirmed by comparing each peak’s retention time with that of injected reference standards (Sigma-Aldrich, St. Louis, MI, USA).

The determination of immunoglobulin A, G, and M was by RP-HPLC chromatography (Series 1100; Agilent Technologies Waldbronn, Germany), according to the methodology described by Puppel et al. [67]. Separations were performed at ambient temperature using solvent gradient with a C18 300A Jupiter column (Phenomenex, Torrance, CA, USA). The identification of peaks was confirmed by comparing each peak’s retention time with that of injected reference standards (Sigma-Aldrich, St. Louis, USA).

The determination of fat-soluble vitamins and β-carotene was by RP-HPLC chromatography (Series 1100; Agilent Technologies Waldbronn, Germany). Separations were performed at ambient temperature using solvent gradient: solvent A was methanol (Merck, Darmstadt, Germany), water (Sigma-Aldrich, St. Louis, MI, USA) in a ratio of 100:900 (*v*/*v*); solvent B was water, methanol in a ratio of 900:100 (*v*/*v*), with a ZORBAX Eclipse XDB column (Agilent Technologies, Waldbronn, Germany). The total run time was 7 min, the flow rate was 1.2 mL/min, and the detection wavelength was 280 nm. The identification of peaks was confirmed by comparing each peak’s retention time with that of injected reference standards (Sigma-Aldrich, St. Louis, MI, USA).

Fatty acid methylation was performed according to the trans-esterification method EN ISO 5509 [68]. Individual fatty acids were identified in crude fat using an Agilent 7890A GC (Agilent, Waldbronn, Germany), according to Puppel et al. [68]. Each peak was identified using pure methyl ester standards: FAME Mix RM–6, Lot LB 68242; Supelco 37 Comp. FAME Mix, Lot LB 68887; Methyl linoleate, Lot 094K1497; CLA Conjugated (9Z, 11E), Lot BCBV3726 (Supelco, Bellefonte, PA, USA). 

### 4.3. Statistical Analyses

The data obtained were statistically processed using multivariate analysis of variance by the least squares method, using the SPSS 22 software [69]. Significant differences among group means were calculated using the F statistic. The distribution of Immunostimulating components in bovine colostrum was examined using the Shapiro–Wilk test. Only those interactions between factors whose influence was statistically significant (*p* ≤ 0.01 or *p* ≤ 0.05) were included in the study—as determined after preliminary statistical analyses. Data were presented as least squares means with a standard error of the mean.

## 5. Conclusions

The experiment showed that supplementation with linseed and fish oil had a significant effect on the basic chemical composition of colostrum. The levels of both protein and fat increased. Supplementation also had a positive effect on the content of immunomodulatory components in the colostrum’s protein fraction. The IgG, IgA, and IgM immunoglobulin content, as well as that of lactoferrin, increased significantly. The addition of a mixture of flaxseed and fish oil also increased the level of vitamins in colostrum, especially vitamin A. The supplementation used had a beneficial effect on the fatty acid profile. The DHA and CLA contents increased, while the LA and AA contents decreased. However, the ALA content changed in an unfavorably way. The study showed that the experimental group receiving LC showed a significantly lower reduction in immunoglobulin concentration over the course of the experiment. A similar relationship was also shown for the formation of fatty acids and fat-soluble vitamins in the LC group and collection 1a, 1b, 2a, 2b, 3, 4, and 5. Therefore, it can be concluded that the supplementation used had a positive effect on the quality of colostrum/milk during the first five days of lactation, stabilizing its composition.

Due to the lower quality of colostrum found in high-yield cows, and therefore in the Holstein–Friesian breed, it is possible to improve the quality by, among other things, introducing nutritional modifications during the second stage of the dry period.

## Figures and Tables

**Table 1:** Daily rations of the cows.

Feed (kg/cow/day)	Dry Cows Groups			
	I (First 5 Weeks)		II (Last 3 Weeks)	
	Dry Matter (kg)	Feed (kg)	Dry Matter (kg)	Feed (kg)
**Roughage:**	**12.12**	**24.20**	**9.25**	**23.50**
Maize silage			4.65	16.00
Alfalfa silage			3.05	5.55
Grass silage	7.65	19.55		
Corn grain			0.42	0.87
Straw	3.26	3.69	1.20	1.30
**Concentrates:**	**0.65**	**0.65**	**2.48**	**2.80**
Fodder chalk			0.05	0.05
Prophos Trans	0.15	0.15	0.15	0.15
Rape meal	0.45	0.50	0.53	0.60
Soya meal			0.92	1.20
Grain meal			0.86	1.10

**Table 2:** Fatty acid composition of fish oil and linseed.

Fatty Acid	Fish Oil [g/kg]	Linseed [g/kg]
C14:0	47.5	0.4
C18:0	2.5	42.7
C18:1 cis-9	251.4	167.7
C18:2n-6	42.3	138.9
C18:3n-3	31.1	561.6
C20:5n-3	73.2	0.00
C22:5n-3	7.7	0.00
C22:6n-3	134.4	0.2

## Data Availability

All data generated or analyzed during the study are included in this published article. The datasets used and/or analyzed in the current study are available from the corresponding author on reasonable request.
